# Abnormal power and spindle wave activity during sleep in young smokers

**DOI:** 10.3389/fnins.2025.1534758

**Published:** 2025-02-11

**Authors:** Youwei Dong, Yongxin Cheng, Juan Wang, Zhiwei Ren, Yiming Lu, Kai Yuan, Fang Dong, Dahua Yu

**Affiliations:** ^1^School of Digital and Intelligent Industry (School of Cyber Science and Technology), Inner Mongolia University of Science and Technology, Baotou, Inner Mongolia, China; ^2^Life Sciences Research Center, School of Life Science and Technology, Xidian University, Xi’an, Shaanxi, China; ^3^School of Automation and Electrical Engineering, Inner Mongolia University of Science and Technology, Baotou, Inner Mongolia, China

**Keywords:** smoking, sleep spindle wave, polysomnography, electroencephalography, power analysis

## Abstract

**Introduction:**

Smoking is associated with significant alterations in sleep architecture. Previous studies have revealed changes in the subjective sleep of young smokers, but research on objective sleep assessment using polysomnography (PSG) is limited. This study aims to explore electroencephalography (EEG) power and sleep spindle activity during the sleep of young smokers, as well as to assess the relationship between sleep and smoking variables.

**Methods:**

We collected overnight PSG data from 19 young smokers and 16 non-smokers and assessed nicotine dependence and cumulative effects using the Fagerstrom Nicotine Dependence Test (FTND) and pack-year. Power spectral analysis and sleep spindle detection are used to analyze EEG activity during sleep.

**Results:**

Compared to the non-smokers, young smokers showed increased alpha power in the frontal and central regions and decreased delta power in the central region. The frontal region showed enhanced sleep spindle duration and density. Notably, both relative alpha power and sleep spindle duration in frontal showed a positive correlation with Pack-year.

**Discussion:**

Sleep EEG power and sleep spindle activity in frontal may serve as biomarkers to assess the sleep quality of young smokers. It may improve the understanding of the relationship of sleep and smoking.

## 1 Introduction

The 2023 Global Tobacco Epidemic Report pointed out that tobacco use remains one of the greatest public health threats, causing over 8 million deaths annually (Organization, W.H, 2023). Smoking, as a leading preventable cause of death, is associated with an increased risk of cardiovascular diseases and cancer (Ambrose and Barua, 2004; Sasco et al., 2004; Wen et al., 2023). Previous Electroencephalogram (EEG) studies found that smoking may affect EEG activity (Yin et al., 2016; Dong et al., 2021; Li et al., 2022; Wang et al., 2022; Wang et al., 2024). For example, the increased alpha coherence between the frontal lobes in young smokers was related to inhibitory control (Wang et al., 2022). The reduced resting-state EEG power in young smokers was associated with poorer performance on inhibitory control tasks (Dong et al., 2021). Therefore, studying young smokers is essential to understanding the impact of smoking on brain development and health outcomes.

Recent studies have shown that smoking may affect sleep quality and brain activity during sleep (Truong et al., 2021; Grigoriou et al., 2024). Subjective and objective sleep quality are important in evaluating sleep quality (Stanyer et al., 2021). Studies based on the Pittsburgh Sleep Quality Index (PSQI) showed that smokers had poorer subjective sleep quality (Grigoriou et al., 2024). Polysomnography (PSG) is the gold standard for assessing objective sleep structure, which includes electroencephalography (EEG), electromyography (EMG), and electrooculography (EOG; Burchard and Chidekel, 2024). By visually scoring the PSG data and classifying sleep stages, the macro and micro structures of sleep were further analyzed (Truong et al., 2021). Previous PSG studies showed that smokers may exhibit alterations in their macro sleep structure, including reduced rapid eye movement (REM) sleep, increased N1 and N2 sleep, prolonged sleep onset latency (SOL) and wake time after sleep onset (WASO; Zhang et al., 2006; Grigoriou et al., 2024).

Moreover, smoking may influence the microstructure of sleep, such as EEG power and spindle activity during sleep (O'Reilly et al., 2019; Truong et al., 2021). During NREM sleep, smokers exhibited decreased delta power and increased alpha power, which closely resemble the EEG patterns observed in individuals with insomnia (Truong et al., 2021; Zhao et al., 2021; Guo et al., 2023). PSG studies in young nonsmokers have shown that transdermal nicotine affects sleep spindle activity (O'Reilly et al., 2019). The stimulant effects of nicotine are important potential factors influencing sleep architecture, including the impact on neurotransmitter systems and melatonin secretion. Previous studies demonstrated that nicotine binds to nicotinic acetylcholine receptors (nAChRs) in the brain, leading to the release of neurotransmitters (Costa and Esteves, 2018; von Deneen et al., 2022; Wen et al., 2024; Wen et al., 2025). This process likely enhanced wakefulness and reduced sleep depth and may have influenced neural circuits related to brain reward systems, potentially altering motivation and behavior (Zhang et al., 2006; Yu et al., 2017; Wang et al., 2019). Melatonin secretion played a critical role in facilitating sleep onset and regulating the sleep–wake cycle (Zisapel, 2018). By stimulating neurotransmitter release, nicotine may have disrupted melatonin secretion, thereby affecting sleep architecture (Georgakopoulou et al., 2024).

Compared to middle-aged and older smokers, there are fewer studies on the sleep of young smokers (Patterson et al., 2018; Pataka et al., 2021; Truong et al., 2021). Young adults are aged between early and middle adulthood. During this time, the sleep patterns is changing and may be affected by the nicotine in cigarettes (Li et al., 2018; Purani et al., 2019). Our aim is to investigate both the macro and micro sleep structures of young smokers and analyze the relationship between sleep structure changes and smoking-related variables. We hypothesized that the sleep structure in young smokers were changed compared with non-smokers, which may be correlated with smoking characteristics.

## 2 Materials and methods

### 2.1 Participants

Nineteen young smokers (mean age: 20.43 ± 1.03 years) and 16 matched non-smokers (mean age: 19.88 ± 1.03 years) were included in this study. All participants were the undergraduate students of Inner Mongolia University of Science and Technology (IMUST). Therefore, they exhibited similar lifestyle habits and dietary patterns, including regular sleep–wake schedules, academic routines, and eating habits typical of university students. These common characteristics helped minimize variability due to external factors. Every participant was right-handed as measured by the Edinburgh Handedness Questionnaire (Casey, 2015). The smokers were diagnosed with nicotine dependence according to the Diagnostics and Statistical Manual of Mental Disorder-V (DSM-V). Non-smokers were recruited by posters during the same period.

Exclusion criteria for both nonsmokers and Smokers included: (1) current use of sedative hypnotics; (2) current diagnosis of major mental conditions (i.e., major depression, major anxiety, schizophrenia), severe physical diseases (i.e., acute or chronic heart, hepatic or renal failure); (3) neurological disorder with changed EEG activities (i.e., Parkinson’s disease, Alzheimer’s disease or seizure disorder); (4) diagnosed with other substance use disorder according to DSM-V.

### 2.2 Procedure

Participants who satisfied both the inclusion and exclusion criteria were invited to participate in this study. Prior to the PSG recording, participants were required to complete a series of standardized questionnaires including Pittsburgh sleep quality index (PSQI), Self-Rating Anxiety Scale (SAS), Self-rating depression scale (SDS), Insomnia Severity Index (ISI), Self-Rating Scale of Sleep (SRSS) to evaluate sleep quality and the degree of anxiety and depression (Morin et al., 2011; Dunstan and Scott, 2020; Liu D. et al., 2021). For smokers, we tested the Fagerstrom test for nicotine dependence (FTND) for nicotine dependence and pack-year to assess the cumulative effect of nicotine (Heatherton et al., 1991). Finally, each participant underwent overnight PSG recording. This study was approved by the Research Ethics Committee of the First Affiliated Hospital of Baotou Medical College of IMUST (2020001) and informed consent was obtained for all subjects. No smoking cessation programs, medications, or other interventions were implemented during the study.

### 2.3 Polysomnography

The participants underwent overnight, supervised, laboratory-based video polysomnography. We recorded PSG data during the period from 10 PM to 6 AM. The lights were turned off at 10 PM, and the subjects were instructed to remain in bed and attempt to fall asleep. The subjects woke up at a fixed time, which was 6 AM. For this study, sleep recordings were analyzed on a subset of the recording montage, including frontal (F3, F4), central (C3, C4) and occipital (O1, O2) electrodes, recorded with a vertex reference (Cz) and re-referenced off-line to averaged mastoids. The entire night’s sleep data were divided into multiple 30-s epochs according to American Academy of Sleep Medicine (AASM) standards, and manual sleep stage scoring was performed (Berry et al., 2012). Macro sleep variables were analyzed based on the results of sleep staging. Among them, Time in Bed (TIB) was defined as the total Time from the start of recording to waking up the next day. SPT was defined as the time from the first non-awake stage to the last non-awake stage. WASO is the total recording time of awake phase during SPT. Total Sleep Time (TST) was the recording time of the whole night except the waking stage. Sleep Efficiency (SE) was used to describe the proportion of TST in TIB. SOL is latency to first epoch of any sleep stage except wake stage. Sleep Maintenance Efficiency (SME) was defined as the percentage of TST over SPT. The absolute and relative durations (Percentage of duration of each stage in TST) of N1, N2, N3 and REM were calculated. The N1, N2, N3 and REM latencies are the times from the beginning of the sleep record to the specific sleep stages.

### 2.4 EEG data preprocessing

We chose N2 sleep for EEG data analysis because N2 is the most stable during sleep and has the largest proportion during sleep (Liu S. et al., 2021). EEG data were processed by MNE-python (Gramfort et al., 2013). Raw data were imported and down-sampled to 100 Hz. Bandpass filter between 0.1 and 40 Hz were subsequently applied. Independent component analysis (ICA) was performed using Fast-ICA algorithm. Experienced researchers then visually inspected these components to identify and exclude those related to electromyographic (EMG) and ocular artifacts. The remaining components were used to reconstruct artifact-free EEG signals. The identified artifacts were manually detected and removed (Hyvärinen and Oja, 2000). Finally, the EEG was visually inspected again to remove epochs with excessive noise or artifacts. The impedance of each EEG electrode was kept below 10 kΩ.

### 2.5 EEG data analyses

The multitaper method was used to calculate the power spectral density of artifact-free, continuous, non-overlapping 6-s epochs on EEG electrodes, which was used to compute the relative signal power in typical frequency bands, including delta (0.5–4 Hz), theta (4–8 Hz), alpha (8–12 Hz), sigma (12–16 Hz), and beta (16–20 Hz). The resulting power values were averaged across different epoch and normalized to the total signal power (0.5–20 Hz) for inter-group comparisons. The multitaper method used multiple discrete prolate spheroidal sequence (DPSS) tapers to average the power spectrum obtained from the EEG signal, which can better reduce the bias and variance of spectrum estimation compared with the Welch method and other single taper methods (Prerau et al., 2017). Automatic spindle wave detection method was adopted for sleep spindle detection (Lacourse et al., 2019). Specifically, the 1-30 Hz EEG signal (EEGbf) and the 12–16 Hz EEG signal (EEGσ) were obtained by FIR filter. Sleep spindles were identified by calculating the relative power of EEGσ in EEGbf and the moving Pearson correlation coefficient and Root mean square (RMS) of EEGσ in EEGbf with a sliding window of 300 ms and a step size of 100 ms. Finally, we calculated the spindle characteristics, including the average amplitude, duration, and density (spindles per minute, spm) of spindles in the range of 12 to 16 Hz for each EEG channel.

### 2.6 Statistical analysis

First, Shapiro–Wilk test was used to determine the normality of the data before analysis. According to the normality results, Welch-t test or Mann–Whitney U test was used to analyze the differences of sleep variables or scale scores. We controlled the false discovery rate by FDR correction. The correlation between macro sleep variables, scale scores and EEG activity during sleep were analyzed by Pearson’s correlation coefficient or Spearman’s correlation coefficient. *p* < 0.05 was considered statistically significant.

## 3 Results

### 3.1 Demographics and scores on the subjective scale

Demographic variables and scale scores are presented in Table 1. No significant difference in age and education level were found between young smokers and non-smokers. In the subjective scale scores, although young smokers showed a decreasing trend in PSQI scores (*p* = 0.056), there were no significant difference between young smokers and non-smokers across all scale scores, including assessments related to sleep status as well as anxiety and depressive mood evaluations.

**Table 1:** Demographic variables and subjective scale scores.

	Smokers	Non-smokers	*p*-value
Demographic variables			
Age(years)	20.4 ± 1.1	19.5 ± 1.7	0.3
Sex	Male	Male	–
Education(years)	14.4 ± 0.77	15.1 ± 1.4	0.3
Pack-year	1.4 ± 0.94	–	–
Subjective scale scores			
PSQI	5.3 ± 2.7	4.1 ± 2.1	0.056
ISI	17.2 ± 3.8	6.3 ± 4.3	0.11
SRSS	19.4 ± 4.2	3.7 ± 4.6	0.15
SAS	37.9 ± 7.6	44.0 ± 9.1	0.21
SDS	39.8 ± 9.8	47.2 ± 13.1	0.36
FTND	2.4 ± 1.6	–	–

Values are expressed as means ± standard deviations. PSQI, pittsburgh sleep quality index; ISI, insomnia severity index; SRSS, self-rating scale of sleep; SAS, self-rating anxiety scale; SDS, self-rating depression scale; FTND, fagerstrom test for nicotine dependence.

### 3.2 Association between sleep characteristics and smoking status

Table 2 shows the relationship between macro sleep structure and smoking status. Among young individuals, smokers had longer N1 latency (mean: 37.6 ± 51.6 min; *p* < 0.05) and SOL (mean: 24.3 ± 17.6 min; *p* < 0.05), as well as reduced N2 sleep (mean: 150.8 ± 38.9 min; *p* < 0.05) and SPT (mean: 385 ± 66.0 min; *p* < 0.05) compared to non-smokers. The sleep latency of N2 and N3 exhibited similar trends to the N1 latency. The proportions of each sleep stage, SE, and SME showed no significant differences between young smokers and non-smokers.

**Table 2:** Multivariable associations between sleep macrostructure and smoking status.

Macro sleep variables	Smoker	Non-smoker	*p*-value
TIB (min)	420.3 ± 74.4	459.0 ± 73.7	0.133
SPT (min)	385.8 ± 66.0	438.7 ± 74.1	0.035
WASO (min)	62.5 ± 33.3	72.5 ± 48.3	0.788
TST (min)	323.3 ± 61.5	366.1 ± 77.3	0.084
N1 (min)	18.1 ± 11.9	22.1 ± 17.03	0.665
N2 (min)	150.8 ± 38.9	183.5 ± 41.11	0.023
N3 (min)	77.3 ± 20.9	79.7 ± 25.73	0.764
SOL (min)	24.3 ± 17.6	13.8 ± 8.532	0.029
Latency of N1 (min)	37.6 ± 51.6	19.1 ± 22.35	0.031
Latency of N2 (min)	34.5 ± 22.7	21.5 ± 15.33	0.067
Latency of N3 (min)	49.1 ± 41.1	27.8 ± 14.95	0.053
Latency of REM (min)	74.1 ± 45.4	90.8 ± 47.3	0.298
REM (%)	23.5 ± 4.34	22.1 ± 4.9	0.402
NREM (%)	76.5 ± 4.34	77.8 ± 4.95	0.402
N1 (%)	5.6 ± 3.59	5.84 ± 3.64	0.857
N2 (%)	46.6 ± 7.78	50.2 ± 6.941	0.155
N3 (%)	24.2 ± 6.18	21.7 ± 4.938	0.193
SME (%)	83.9 ± 7.47	83.3 ± 11.76	0.806
SE (%)	77.4 ± 9.78	79.6 ± 11.55	0.385

Values are expressed as means ± standard deviations. TIB, time in bed; SPT, sleep period time; WASO, wake after sleep onset; TST, total sleep time; SE, sleep efficiency; SME, sleep maintenance efficiency.

### 3.3 Association between EEG activity and smoking status

**Figure 1:**
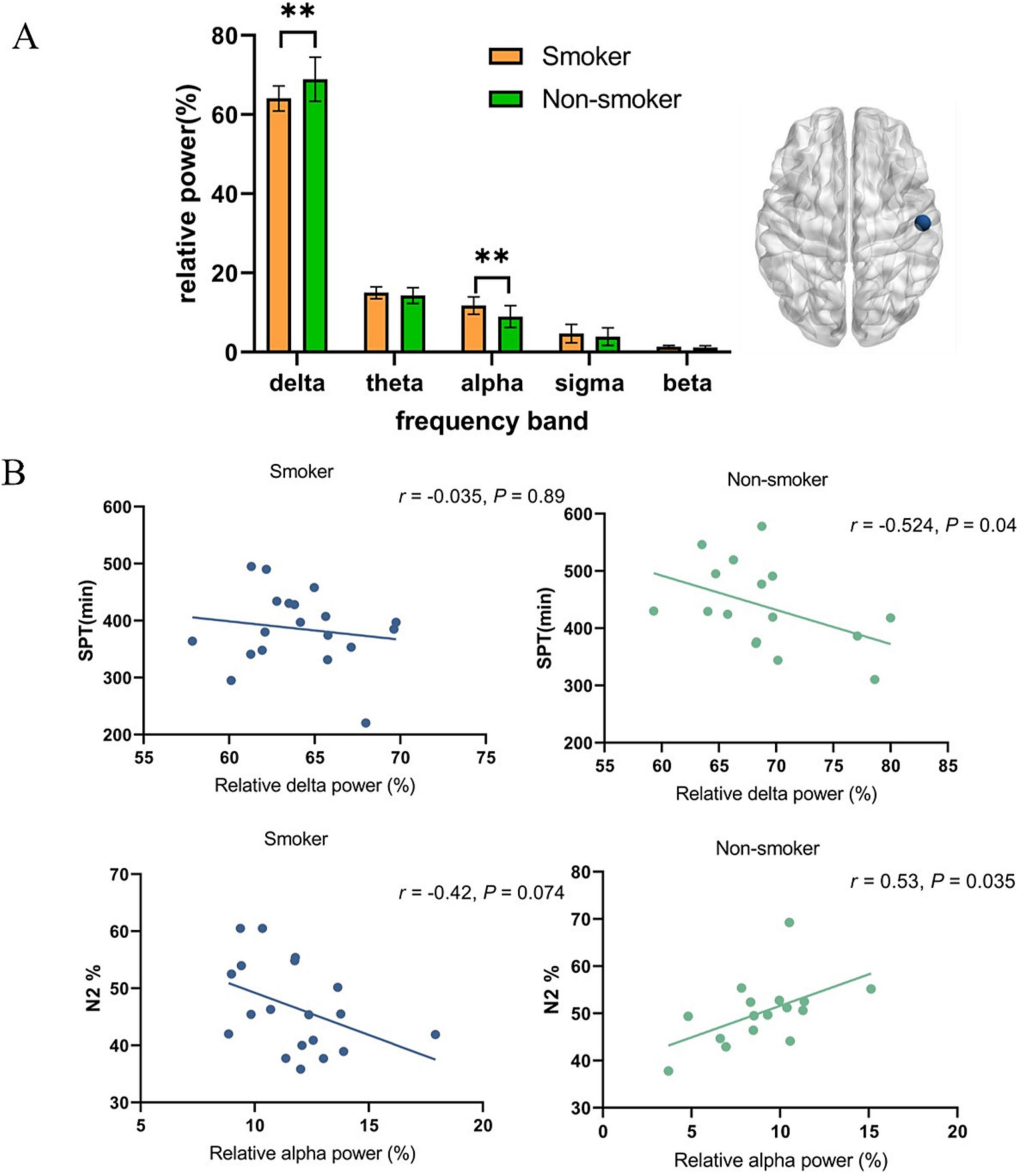
The EEG activity differences at the C4 electrode during N2 sleep. **(A)** Young smokers exhibited a decrease in delta power (*t* = 3.07, *p* < 0.01) and an increase in alpha power (*t* = 3.26, *p* < 0.01). **(B)** Relative delta power did not show a significant correlation with SPT (*r* = −0.035, *p* > 0.05) but it was negatively correlated in non-smokers (*r* = −0.524, *p* = 0.04). Relative alpha power at C4 electrode was positively correlated with N2% (*r* = 0.53, *p* = 0.035), while it was disappeared in young smokers (*r* = −0.419, *p* = 0.074). *p < 0.05, **p < 0.01.

**Figure 2:**
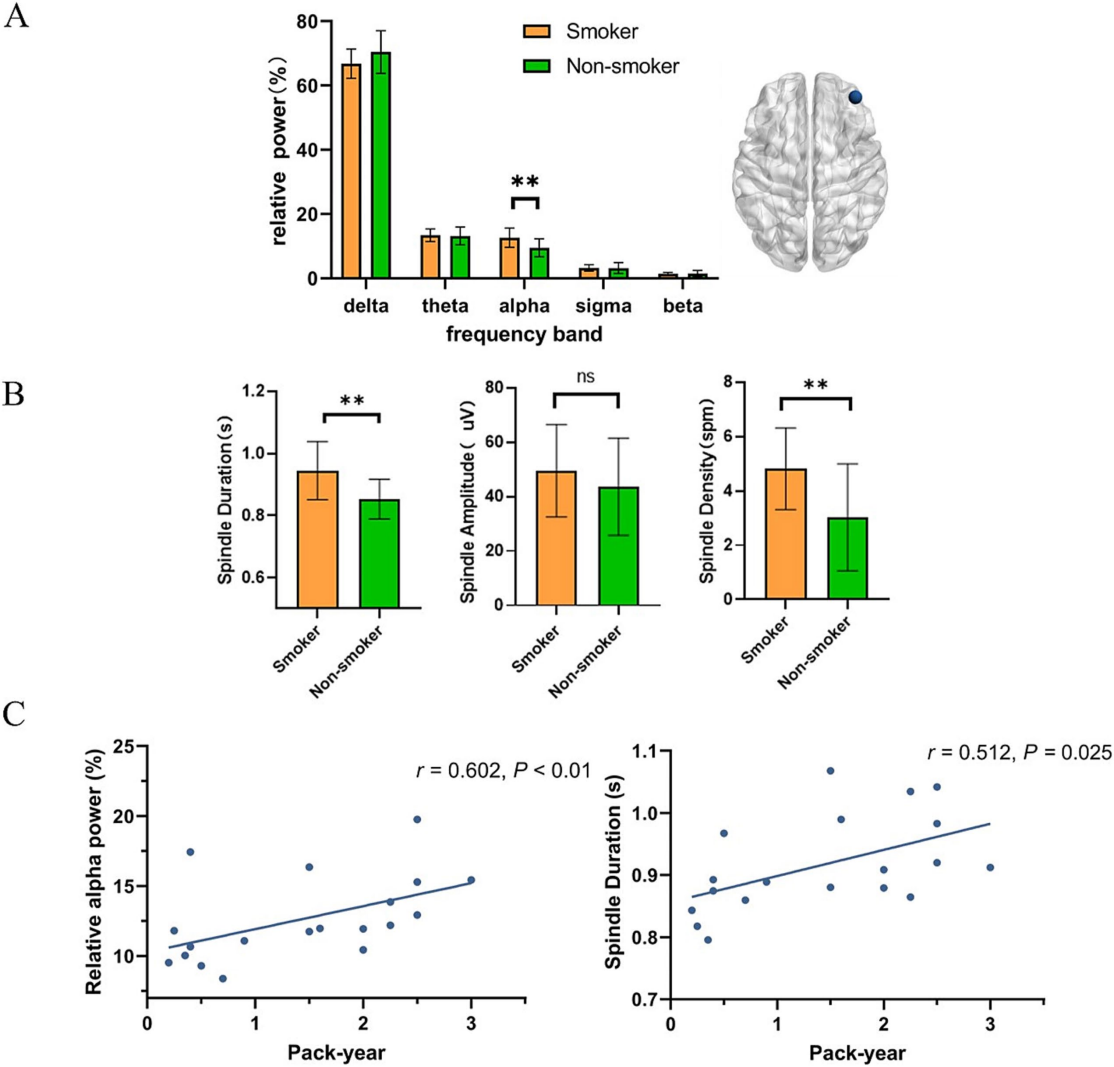
The association between EEG activity at the F4 electrode during N2 sleep. **(A)** Compared to young non-smokers, alpha power was higher in smokers (*U* = 59.0, *p* < 0.01). **(B)** Young smokers exhibited higher spindle density (mean spindle density:4.8 spm, 95% confidence intervals (CI): [0.55, 3.01], Hedges’g = 0.49, *p* < 0.01) and longer duration (mean spindle duration: 0.94 s, 95% CI: [0.04, 0.14], Hedges’g = 0.50, *p* < 0.01). **(C)** Relative alpha power in young smokers was positively correlated with pack-year (*r* = 0.602, *p* < 0.01). The duration of sleep spindles in young smokers was positively correlated with pack-year. (*r* = 0.512, *p* = 0.025). *p < 0.05, **p < 0.01.

Figures 1, 2 present the EEG relative power under different smoking status. We found that young smokers exhibited a decrease in delta power (*t* = 3.07, *p* < 0.01) and increase in alpha power (*t* = 3.26, *p* < 0.01) at C4 electrode during N2 sleep. No significant differences were found in the other three frequency bands (theta, sigma, beta). Additionally, significant increase in alpha power was also observed at F4 electrode in young smokers (*U* = 59.0, *p* < 0.01). The activity of sleep spindle waves under different smoking statuses is illustrated in Figure 2. Young smokers exhibited greater spindle density (*U* = 64.0, Hedges’g = 0.49, *p* < 0.01) and longer duration (*U* = 63.0, Hedges’g = 0.50, *p* < 0.01) compared to non-smokers at F4. No significant inter-group differences were found in spindle amplitude.

Additionally, we investigated the relationship between micro-sleep structure and macro-sleep structure. At C4 electrode, relative delta power did not show significant correlation with SPT (*r* = −0.035, *p* > 0.05) but it was negatively correlated in non-smokers (*r* = −0.524, *p* = 0.04). Similarly, we also found that in young non-smokers, relative alpha power at the C4 electrode was positively correlated with the proportion of N2 sleep (*r* = 0.53, *p* = 0.035) but no significant correlation in young smokers. (*r* = −0.419, *p* = 0.074).

Finally, we investigated the relationship between smoking variables (pack-year and FTND) and microstructure of sleep. Through correlation analysis, we found that relative alpha power at F4 electrode in young smokers was positively correlated with pack-year (*r* = 0.602, *p* < 0.01). The duration of sleep spindles at F4 electrode in young smokers was positively correlated with pack-year. (*r* = 0.512, *p* = 0.025).

## 4 Discussion

In this study, we used PSG to assess sleep quality in young smokers and the relationship between sleep variables and smoking-related variables. In subjective sleep assessments, we failed to find significant different PSQI scores in young smokers compared with non-smokers but it exhibited a rising trend as *p =* 0.056. Similar to previous studies, results of macro sleep structure indicated that young smokers had reduced SPT, decreased N2 sleep and prolonged SOL and N1 latency (Yosunkaya et al., 2021; Mauries et al., 2023).

Young smokers showed changes in sleep microstructure, including reduced delta power and increased alpha power at C4 electrode. An enhancement in alpha power at F4 electrode was found, which positively correlated with pack-year. It is noteworthy that enhanced spindle density and prolonged spindle duration in young smokers was observed, which was correlated with pack-year. We explored the association between the macrostructure and microstructure of sleep. In young non-smokers, we found associations between relative power including delta and alpha power and macro sleep structure, which were not observed in young smokers.

Firstly, our study similarly showed that no significant difference of PSQI score in young smokers compared with non-smokers, but we found the trend of the increase of PSQI score. Middle-aged and elderly smokers have poorer subjective sleep quality based on the PSQI questionnaire (Liao et al., 2019; Purani et al., 2019). Single cohort study involving 405 young smokers showed that 36% of young smokers had poor sleep quality (PSQI >5; Dugas et al., 2017). Significant difference of PSQI between young non-smokers and smokers were not reported in other studies (Cohen et al., 2020; Al-Mshari et al., 2022).

Secondly, we found decreased delta power and increased alpha power during N2 sleep in young smokers, which was similar to the findings in insomnia PSG study (Zhao et al., 2021). The effect of sleep by nicotine may be a reason for changes in EEG power during sleep (Saint-Mleux et al., 2004; Sharma et al., 2015). Transdermal nicotine patches may increase alpha power during first NREM-REM sleep cycle and decrease the delta power during N2 sleep in young smokers (Choi et al., 2017). Animal study similarly showed that nicotine indirectly inhibited the sleep-promoting neurons in the ventrolateral preoptic area while directly activating neurons related to the arousal system (Saint-Mleux et al., 2004). Additionally, we observed a significant negative correlation between relative delta power during NREM sleep and SPT in non-smokers. In the two-process model of sleep regulation, EEG delta power was often used as an indicator of the S process, reflecting the release and recovery of sleep pressure (Davis et al., 2011). The correlation between delta power and SPT might have reflected the homeostatic regulation of sleep pressure, suggesting that the brain required higher delta activity when sleep pressure had not been sufficiently released. Previous studies demonstrated that both delta power during NREM sleep and the duration of NREM sleep increased following sleep deprivation (Achermann, 2004; Long et al., 2021). The regulation of sleep was known to involve multiple neurotransmitters, including acetylcholine and gamma-aminobutyric acid (Jones, 2020). In smokers, this association might have been attenuated due to the stimulant effects of nicotine and its influence on neurotransmitter systems.

Finally, during N2 sleep, we observed an increase in spindle duration and density at the F4 electrode in young smokers, which was positively correlated with pack-year. Sleep spindles are generated by thalamic reticular neurons and thalamocortical neurons, which are extensively projected to the cortex and hippocampus (Andrillon et al., 2011; Fernandez and Lüthi, 2020). Previous studies showed that nAChRs were related to sleep spindles (Ozaki et al., 2012; Ni et al., 2016). The use of acetylcholinesterase inhibitors can restore sleep spindle waves in patients with neurodegenerative diseases (Ozaki et al., 2012). Animal study showed that sleep spindles could be generated by activating nAChRs in the thalamic reticular nucleus (Ni et al., 2016). Our study showed that the spindle activity in young smokers was enhanced and correlated with pack-year. The interaction between nAChRs and sleep spindles supplied a possible explanation for the enhancement of the spindle activity in young smokers: nicotine binds to nAChRs, which increases the activity of sleep spindles in young smokers. In fact, young non-smokers show enhanced spindle activity after transdermal nicotine administration (O'Reilly et al., 2019). It indicated that changes of sleep spindle activity in young smokers may be related to nicotine in tobacco rather than other substances in tobacco.

## 5 Limitation

In this study, a comprehensive analysis of the subjective and objective sleep quality of young smokers was conducted. However, our study still has some limitations. First, the sample size in our study is relatively small. Second, in our participant selection, we focused solely on male smokers. In future research, we will continue to investigate the long-term effects of sleep spindle activity in young smokers on memory consolidation and emotional regulation, which will contribute to a better understanding of the relationship between smoking, sleep, and cognitive performance.

## 6 Conclusion

This study mainly focused on the macro and micro sleep structures of young smokers and the association between sleep structure and smoking. Sleep EEG power and spindle activity may assess sleep quality in young smokers, which may provide new insights into the relationship between smoking and sleep.

## Data Availability

The original contributions presented in this study are included in the article/Supplementary material; further inquiries can be directed to the corresponding authors.
